# Prediction of P-tau/Aβ42 in the cerebrospinal fluid with blood microRNAs in Alzheimer’s disease

**DOI:** 10.1186/s12916-021-02142-x

**Published:** 2021-11-15

**Authors:** Longfei Jia, Min Zhu, Jianwei Yang, Yana Pang, Qi Wang, Ying Li, Tingting Li, Fangyu Li, Qigeng Wang, Yan Li, Yiping Wei

**Affiliations:** Innovation Center for Neurological Disorders and Department of Neurology, Xuanwu Hospital, Capital Medical University, National Clinical Research Center for Geriatric Diseases, 45 Changchun St, Beijing, China

**Keywords:** Alzheimer’s disease, Dementia, MicroRNA, Biomarker, Diagnosis

## Abstract

**Background:**

The most common biomarkers of Alzheimer’s disease (AD) are amyloid β (Aβ) and tau, detected in cerebrospinal fluid (CSF) or with positron emission tomography imaging. However, these procedures are invasive and expensive, which hamper their availability to the general population. Here, we report a panel of microRNAs (miRNAs) in serum that can predict P-tau/Aβ42 in CSF and readily differentiate AD from other dementias, including vascular dementia (VaD), Parkinson disease dementia (PDD), behavioral variant frontotemporal dementia (bvFTD), and dementia with Lewy body (DLB).

**Methods:**

RNA samples were extracted from the participant’s blood. P-tau/Aβ42 of CSF was examined for diagnostic purposes. A pilot study (controls, 21; AD, 23), followed by second (controls, 216; AD, 190) and third groups (controls, 153; AD, 151), is used to establish and verify a predictive model of P-tau/Aβ42 in CSF. The test is then applied to a fourth group of patients with different dementias (controls, 139; AD,155; amnestic mild cognitive impairment [aMCI], 55; VaD, 51; PDD, 53; bvFTD, 53; DLB, 52) to assess its diagnostic capacity.

**Results:**

In the pilot study, 29 upregulated and 31 downregulated miRNAs in the AD group were found. In Dataset 2, these miRNAs were then included as independent variables in the linear regression model. A seven-microRNA panel (miR-139-3p, miR-143-3p, miR-146a-5p, miR-485-5p, miR-10a-5P, miR-26b-5p, and miR-451a-5p) accurately predicted values of P-tau/Aβ42 of CSF. In Datasets 3 and 4, by applying the predicted P-tau/Aβ42, the predictive model successfully differentiates AD from controls and VaD, PDD, bvFTD, and DLB.

**Conclusions:**

This study suggests that the panel of microRNAs is a promising substitute for traditional measurement of P-tau/Aβ42 in CSF as an effective biomarker of AD.

**Supplementary Information:**

The online version contains supplementary material available at 10.1186/s12916-021-02142-x.

## Background

Amyloid β (Aβ) and tau pathologies are classic characteristic features of Alzheimer’s disease (AD), and they are widely used as diagnostic biomarkers [1]. Aβ and tau burden in the brain can be identified with high accuracy from cerebrospinal fluid (CSF) testing [2] and positron emission tomography (PET) imaging [3, 4]. However, the high cost and low availability of PET scans hamper the feasibility of their use in clinical diagnostic practice and clinical trials. Aβ and tau in samples of CSF obtained from patients has been shown to diagnose AD with excellent accuracy [5]. Multiple studies have suggested that the combined measurements of phosphorylated-tau (P-tau) and Aβ42 in the CSF can inform a more accurate diagnosis than either test alone; this improved diagnostic accuracy is likely due to the reduced impact of preanalytical and analytical confounders [6–8]. Further supporting the CSF ratio of P-tau/Aβ42 as a reliable diagnostic biomarker for AD, several studies have reported similar threshold levels, in the range of 0.09–0.14 [7, 9–11]. However, the relatively invasive nature of CSF collection restricts its use as a screening tool in the elderly population. Hence, there is an unmet need for a minimally invasive, widely available, and cost-effective method of measuring biomarkers for the early detection of AD in the general population.

By measuring a panel of microRNAs (miRNAs) in the blood, the current study proposes a simple, antibody-independent method of predicting the P-tau/Aβ42 ratio in the CSF. miRNAs are short non-coding RNAs of approximately 20–25 nucleotides in length that bind to complementary sites on the 3′ untranslated region (UTR) of their mRNA targets, curbing their expression [12]. Changes in miRNA expression may induce translational abnormalities, resulting in the alteration of corresponding protein levels. An increasing number of studies have demonstrated a relationship between miRNAs and AD; by targeting the expression of amyloid precursor protein (APP) or beta-site APP cleaving enzyme 1 (BACE1) [13], miRNAs can directly affect potential pathogenic pathways and thus alter the risk and/or progression of AD. A panel of 12 miRNAs can reportedly diagnose AD with high performance [14], indicating the combination of miRNA panels as a promising biomarker for AD. However, a recent review paper listed 48 studies on circulating microRNAs as potential biomarkers for AD, which showed inconsistent data [15]. The first potential reason of the inconsistencies among these studies may be the small sample sizes of these studies. The sample size of these studies ranged from 6 to 287 (AD patients), and 29 studies (over 60.4% of 48 studies in total) included a sample size of < 30 AD patients. The too-small-sample-size studies may produce bias in the results. The second reason for the inconsistencies would be that most studies did not use CSF or PET biomarkers to recruit AD patients. In this study, the strict inclusion criteria involving CSF biomarkers and a large sample size were recruited, which may guarantee the potential clinical application of positive findings.

In addition, AD and other types of dementia, such as vascular dementia (VaD), Parkinson disease dementia (PDD), behavioral variant frontotemporal dementia (bvFTD), and dementia with Lewy body (DLB), may have overlapping clinical manifestations, pathology, and biomarkers, often resulting in difficulties in clinical diagnosis [16]. Whether miRNAs can differentiate AD from other forms of dementia has been addressed by few studies. Given the crucial role of miRNAs in the expression of genes that are key to AD pathology, their relative stability, tissue enrichment, and amenability to quantitative measurement [15], we speculated that measuring single or multiple miRNAs may reflect the concentration of Aβ and tau in the brains of AD patients. Therefore, this study aimed to evaluate whether the levels of blood miRNAs (1) predict the P-tau/Aβ42 ratio in the CSF, (2) can be used to differentiate patients with AD from cognitively normal controls, and (3) can effectively discriminate AD from VaD, PDD, bvFTD, and DLB.

## Methods

### Experimental design

**Fig. 1:**
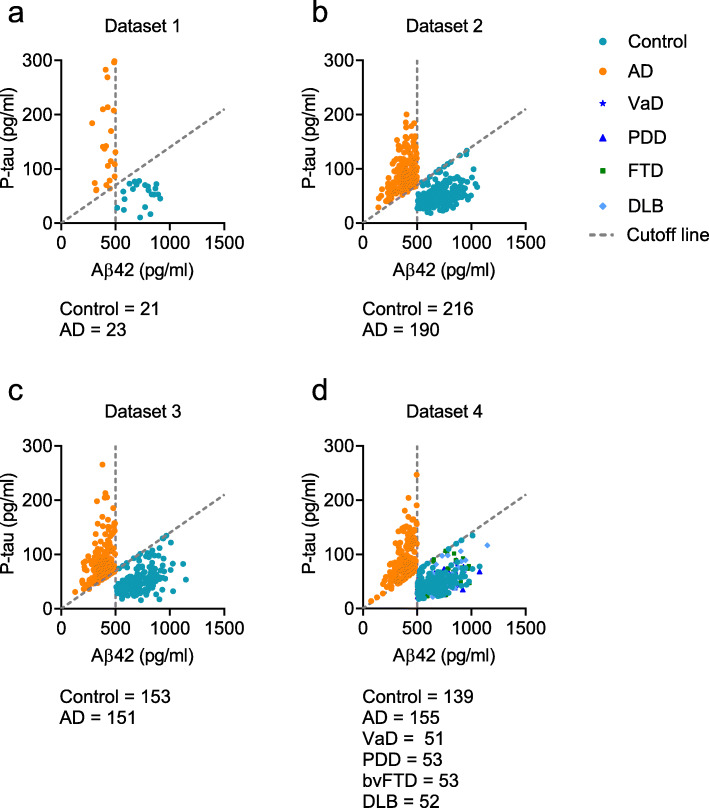
The levels of Aβ42 and P-tau in the cerebrospinal fluid of cognitively normal controls and patients with Alzheimer’s disease (AD). The cutoff value of P-tau/Aβ42 (0.14) was used to determine AD in Dataset 1 (**a**), 2 (**b**), 3 (**c**), and 4 (**d**). Dotted line: slope = 0.14 indicates the cutoff value of P-tau/ Aβ42; 500 pg/ml in horizontal axis indicates the cutoff value of Aβ42. Abbreviations: AD, Alzheimer’s disease; VaD, vascular dementia; PDD, Parkinson disease dementia; bvFTD, behavioral variant frontotemporal dementia; DLB, dementia with Lewy body

Four datasets were acquired in this study (Fig. 1, Tables 1, 2, 3, and 4). The data for the pilot study (Dataset 1) were obtained from a Beijing center (*n* = 44; controls, 21; AD, 23); those for the development of our model (Dataset 2) were collected from centers in the provinces of Shandong, Henan, and Guangxi (*n* = 406, controls, 216; AD, 190); those for the validation of the model (Dataset 3) were acquired from centers in Guizhou, Hebei, Jilin, and the Inner Mongolia Autonomous Region (*n* = 304; controls, 153; AD, 151). Those for the application of the model (Dataset 4) were acquired from Beijing center (*n* = 503; control, 139; AD, 155; amnestic mild cognitive impairment [aMCI], 55; VaD, 51; PDD, 53; bvFTD, 53; DLB, 52). Diagnoses of AD were based on the criteria published by the National Institute on Aging and Alzheimer’s Association (NIA-AA) [1]. Informed by previously published data [10, 11], a cutoff value for the P-tau/Aβ42 ratio of 0.14 was used to differentiate patients with AD from normal controls (Fig. 1a–d). In addition, based on the ATN framework, low CSF Aβ42 is the key “Alzheimer’s pathological change” [17]. We, therefore, used a reported CSF Aβ42 cutoff of 500 pg/ml (Fig. 1c) [18] as another inclusion criterion. Diagnoses of aMCI [19], VaD [20], PDD [21], bvFTD [22], and DLB [23] were based on previously published criteria. In addition, other neurodegenerative diseases may share overlapping clinical manifestations and pathology with AD, which often result in difficulties in clinical diagnosis. To avoid the mixture of other dementias and AD, patients with VaD, PDD, bvFTD, and DLB who had AD-like cutoff values of P-tau/Aβ42 and Aβ42 were excluded (Fig. 1d, Additional file 1: Fig. S1). A previously published paper has showed the very different levels of CSF P-tau and Aβ42 in VaD, PDD, FTD, and DLB [24]. It would reasonable to use P-tau/Aβ42 and Aβ42 as biomarkers for these diseases. The details of diagnostic criteria were included in Additional file 2: materials and methods. Written informed consent was obtained from all participants or their legal guardians. This study was approved by the Institutional Ethics Board of Xuanwu Hospital, Capital Medical University (LYS[2017]004).

**Table 1:** Characteristics of participants in Dataset 1

Characteristic	Total sample (*n* = 44)	Controls (*n* = 21)	AD (*n* =23)
Age, median (25th–75th percentile)	69 (66–73)	69 (67–72)	72 (65–73)
Education year, median (25th–75th percentile)	9 (7–11)	10 (8–11)	9 (7–10) *
Women, No. (%)	23 (52.3)	12 (52.2)	11 (52.4)
*ApoE* ε4 positive (%)	13 (29.5)	4 (17.4)	9 (42.9) *
MMSE score (SD)	25.2 (4.1)	28.7 (0.8)	22.0 (3.1) *
Aβ42, mean (SD), pg/ml	572.8 (186.4)	740.1 (118.6)	420.1 (63.1)
T-tau, mean (SD), pg/ml	443.3 (206.6)	297.1 (100.2)	576.7 (187.9)
P-tau, mean (SD), pg/ml	104.9 (76.9)	52.9 (20.4)	152.5 (79.2)

The values of age, education year, and MMSE are shown as mean (SD)

Abbreviations: *AD*, Alzheimer’s disease; *VaD*, vascular dementia; *PDD*, Parkinson disease dementia; *bvFTD*, behavioral variant frontotemporal dementia; *DLB*, dementia with Lewy body; *ApoE* ε4, apolipoprotein ε4; *MMSE*, Mini-Mental State Examination; *SD*, standard deviation

*P < 0.05 compared to controls

**Table 2:** Characteristics of participants in Dataset 2

Characteristic	Total Sample (*n* = 406)	Controls (*n* = 216)	AD (*n* =190)
Age, median (25th–75th percentile)	68 (63–71)	67 (63–72)	68 (64–71)
Education year, median (25th–75th percentile)	10 (8–11)	10.0 (9–11)	9 (8–10)*
Women, No. (%)	211 (52.0)	112 (51.9)	99 (52.1)
*ApoE* ε4 positive (%)	120 (29.6)	41 (19.0)	79 (41.6)*
MMSE score (SD)	24.9 (4.7)	29.0 (1.0)	20.3 (2.4)*
Aβ42, mean (SD), pg/ml	555.5 (199.6)	711.3 (130.7)	378.2 (81.3)
T-tau, mean (SD), pg/ml	460.9 (206.1)	382.3 (98.9)	611.7 (192.1)
P-tau, mean (SD), pg/ml	70.6 (33.1)	52.3 (20.6)	91.4 (32.3)

The values of age, education year, and MMSE are shown as mean (SD)

Abbreviations: *AD*, Alzheimer’s disease; *VaD*, vascular dementia; *PDD*, Parkinson disease dementia; *bvFTD*, behavioral variant frontotemporal dementia; *DLB*, dementia with Lewy body; *ApoE* ε4, apolipoprotein ε4; *MMSE*, Mini-Mental State Examination; *SD*, standard deviation

*P < 0.05 compared to controls

**Table 3:** Characteristics of participants in Dataset 3

Characteristic	Total Sample (*n* = 304)	Controls (*n* = 153)	AD (*n* =151)
Age, median (25th–75th percentile)	69 (64–72)	68 (64–72)	69 (65–73)
Education year, median (25th–75th percentile)	10 (8–11)	10 (9–11)	9 (7–10)*
Women, No. (%)	155 (50.9)	78 (50.9)	77 (50.9)
*ApoE* ε4 positive (%)	90 (29.6)	28 (18.3)	62 (41.1)*
MMSE score (SD)	25.0 (4.5)	29.0 (0.6)	20.9 (2.8)*
Aβ42, mean (SD), pg/ml	559.2 (213.4)	742.6 (128.2)	373.4 (79.2)
T-tau, mean (SD), pg/ml	478.7 (212.9)	322.2 (98.6)	637.3 (177.2)
P-tau, mean (SD), pg/ml	73.4 (36.8)	55.7 (24.2)	91.3 (38.7)

The values of age, education year, and MMSE are shown as mean (SD)

Abbreviations: *AD*, Alzheimer’s disease; *VaD*, vascular dementia; *PDD*, Parkinson disease dementia; *bvFTD*, behavioral variant frontotemporal dementia; *DLB*, dementia with Lewy body; *ApoE* ε4, apolipoprotein ε4; *MMSE*, Mini-Mental State Examination; *SD*, standard deviation

*P < 0.05 compared to controls

**Table 4:** Characteristics of participants in Dataset 4

Characteristic	Total Sample (*n* = 503)	Controls (*n* = 139)	AD (*n* =155)	aMCI (*n* =55)	VaD (*n* =51)	PDD (*n* =53)	bvFTD (*n* =53)	DLB (*n* =52)
Age, median (25th–75th percentile)	67 (63–72)	67 (65–73)	68 (63–73)	67 (64–70)	67 (63–70)	68 (64–73)	66 (62–70)	66 (62–70)
Education year, median (25th–75th percentile)	9 (8–11)	10 (9–11)	9 (8–10)	10 (8–12)	10 (8–11)	9 (8–10)	10.0 (9–11)	9.0 (7–11)
Women, No. (%)	255 (50.7)	71 (51.1)	79 (51.0)	28 (50.1)	24 (47.1)	26 (49.1)	28 (52.8)	27 (51.9)
*ApoE* ε4 positive (%)	133 (26.4)	25 (18.0)	64 (41.3)*	15 (27.3)	13 (25.5)*	10 (18.9)	11 (20.8)	10 (19.2)
MMSE score (SD)	23.4 (4.5)	29.1 (0.57)	20.8 (3.2)*	26.4 (0.6)	21.5 (2.9)*	21.0 (3.2)*	21.9 (3.0)*	21.8 (3.6)*
Aβ42, mean (SD), pg/ml	581.2 (184.7)	683.5 (135)	371.6 (80.7)*	507.3 (134.1)*	705 (108.5)	700.6 (127.6)	678.8 (141.5)	667.6 (142.4)
T-tau, mean (SD), pg/ml	449.2 (166.6)	340.1 (93.6)	595.6 (187.1)	474.3 (130.9)	395.5 (91.6)	359.2 (107.8)	448.6 (110.7)	422.6 (113.4)
P-tau, mean (SD), pg/ml	63.2 (31.5)	50.1 (19.5)	90.6 (37.7)*	65.6 (21.8)*	48.9 (17.7)	49 (13.5)	53 (22.6)	53.2 (25.1)

The values of age, education year, and MMSE are shown as mean (SD)

Abbreviations: *AD*, Alzheimer’s disease; *VaD*, vascular dementia; *PDD*, Parkinson disease dementia; *bvFTD*, behavioral variant frontotemporal dementia; *DLB*, dementia with Lewy body; *ApoE* ε4, apolipoprotein ε4; *MMSE*, Mini-Mental State Examination; *SD*, standard deviation

*P < 0.05 compared to controls

### RNA collection and sequencing

Blood samples were collected in the morning after a 12-h fast. Twenty milliliters of whole blood were drawn from each subject and stored in a polypropylene tube containing EDTA. The whole-blood samples were immediately processed at the Beijing center (Xuanwu Hospital). At the other centers, the collected samples were immediately centrifuged at 4200×*g* for 10 min at room temperature to obtain the plasma, which was then kept at 4 °C and shipped in dry ice to the Beijing central laboratory within 12 h. Total RNA was isolated using the miRNeasy Serum Kit (Qiagen, USA) following the manufacturer’s instructions. For the preparation of the sequencing library, we used 1 μg of total RNA (quantified with Nano Drop 8000 [Thermo Fisher Scientific, USA] and Agilent 2100 bioanalyzer [Agilent, USA]). Total RNA was purified by electrophoretic separation with 15% urea denaturing polyacrylamide gel electrophoresis (PAGE). Small RNA regions corresponding to the 18–30 nt bands in the marker lane (14–30 ssRNA Ladder Marker, TAKARA, Japan) were excised and recovered. The small 18–30 nt RNAs were ligated to adenylated 3′ adapters annealed to unique molecular identifiers (UMI), followed by the ligation of 5′ adapters. The adapter-ligated small RNAs were subsequently transcribed into cDNA by SuperScript II Reverse Transcriptase (Invitrogen, USA). Several rounds of PCR amplification with PCR Primer Cocktail and PCR Mix were then performed to enrich the cDNA fragments. The PCR products were subsequently purified with PAGE gel, and the recycled products were dissolved in EB solution. The double-stranded PCR products were validated on an Agilent Technologies 2100 bioanalyzer. They were then heat-denatured and circularized with the splint oligo sequence. The single-strand circle DNA (ssCir DNA) was formatted as the final library. The library was amplified with phi29 to generate DNA nanoball (DNB), which has more than 300 copies of one molecule. The DNBs were loaded onto the patterned nanoarray, and single-end 50-base reads were generated with combinatorial Probe-Anchor Synthesis (cPAS). The final ligation PCR products were sequenced using the BGISEQ-500 platform (BGI-Shenzhen, China). Samples for quantitative real-time PCR analyses were added with synthetic *Caenorhabditis elegans* miR cel-miR-39 (Qiagen) after homogenization by QIAzol Lysis reagent, as an external calibration for RNA extraction, reverse transcription, and miRNA amplification [25].

### miRNA data analysis

The raw tags—i.e., the raw sequencing data—were processed according to the following procedure: removal of low-quality tags, removal of tags with 5′-primer contaminants, removal of tags without three primers, removal of tags without insertion, removal of tags with poly A, and removal of tags whose lengths were shorter than 18 nt. After filtration, the remaining tags were mapped to the reference genome NCBI GRCh38 and other databases, including miRbase with Bowtie2 [26]. Cmsearch [27] was performed for Rfam mapping. miRDeep2 [28] was used to predict novel miRNAs by exploring secondary structures. The level of miRNA expression was calculated by counting the absolute numbers of molecules using unique molecular identifiers [29]. Differential expression analysis was performed using the DEseq2 [30]; Q value ≤ 0.001 and the absolute value of Log2Ratio ≥ 1 were used as the default thresholds to judge the significance of the differences in expression.

### Quantitative real-time PCR analyses

Quantitative real-time PCR (qPCR) analyses were performed to confirm the altered miRNAs in the validation study. The miRNA levels were quantified using the NCode™ VILO™ miRNA qRT-PCR kit (Invitrogen, USA) and normalized to synthetic *Caenorhabditis elegans* miR-39-3p. All reactions were triplicated in three independent experiments. The 2^-ΔΔCt^ method was used to calculate miRNA expression [31]. All miRNA primers are listed in Additional file 2: Table S1. To improve experimental precision, triplicates for qPCR were performed. Coefficient of variation (CV) was calculated using standard deviation divided by mean value of a group of replicates. All CV% in the study were lower than 5%, indicating that the data were of high quality (Additional file 2: Table S2).

### Collection of CSF and measurement of Aβ42, T-tau, and P-tau

CSF was collected immediately after the collection of blood samples according to international guidelines [32]. Specifically, 15 mL of CSF were collected from each subject using lumbar puncture while they were positioned in a left lateral position. The participants were monitored for any signs of discomfort for at least 12 h following the lumbar puncture. The CSF samples were centrifuged at 2000×*g* for 10 min at room temperature and stored in a polypropylene tube at − 80 °C. The levels of Aβ42, total-tau (T-tau), and P-tau (tau phosphorylated at Thr 181) in the CSF were then measured using an enzyme-linked immunosorbent assay (ELISA) kits, all of which are listed in Additional file 2: Table S2.

### Statistical analysis

Statistical analyses were performed using SPSS v.22 and Stata 13.0. Datasets 1, 2, and 3 were analyzed independently. Group differences in categorical data, such as sex, clinical subgroups, and p ε4 allele (*ApoE ε4*) carrier distributions, were analyzed with the *χ*^2^ test. Group differences in continuous data, such as the concentrations of biomarkers, were analyzed with Welch’s *t* test or analyses of variance (ANOVAs). In Dataset 1, the false discovery rate (FDR) correction was performed to select the differential miRNAs. *Q* values were used to show the analysis results. In Datasets 2 and 3, the correlative analysis of miRNAs and P-tau/Aβ values was performed using a linear regression model. The tolerance, variance inflation factor (VIF), eigenvalue, and condition index were calculated to assess multicollinearity [33]. After the generation of the linear regression model, the predicted values of P-tau/Aβ42 were calculated with miRNA levels. Receiver operating characteristic (ROC) curves were established using the predicted P-tau/Aβ42 ratio. All tests were two-tailed, and the level of significance was set at *P* < 0.05.

## Results

### Participant characteristics

Four datasets were included (Fig. 1). Tables 1, 2, 3, and 4 list the characteristics of the participants. There were no differences in the ages, or ratio of males/females between the AD and control groups in Datasets 1, 2, 3, and 4. The education years, percentages of ApoE ε4, and findings of the Mini-Mental State Examination (MMSE) differed significantly (*P* < 0.05) between AD patients and controls in Datasets 1, 2, and 3. MMSE scores were also reduced in VaD, PDD, bvFTD, and DLB compared to controls in Dataset 4 (all *P* < 0.05).

### A pilot study

**Fig. 2:**
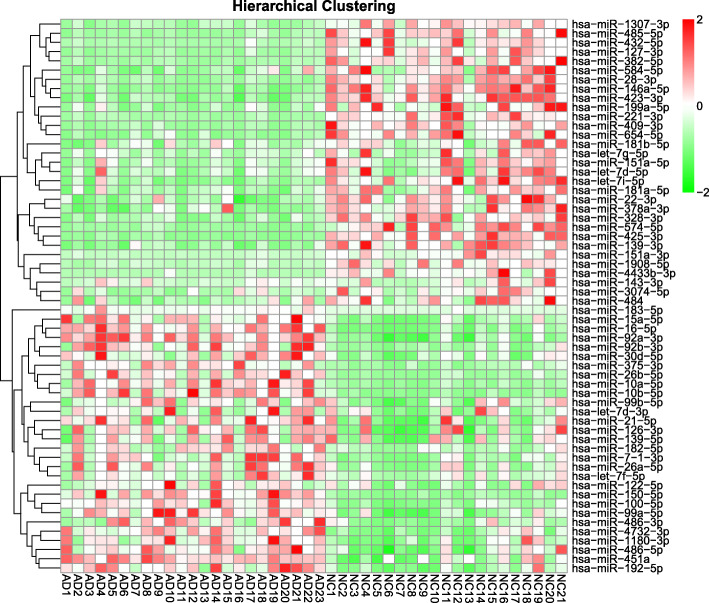
Heat map after hierarchical clustering of the 29 upregulated and 31 downregulated miRNAs in the pilot study (Dataset 1). Abbreviations: miRNA, microRNA; AD, Alzheimer’s disease; NC, normal control

A pilot study was performed in a relatively small sample (Dataset 1). The RNA-sequencing results revealed 860 miRNAs in the blood of patients with AD and the controls. The miRNAs whose read counts were lower than 100 were excluded from subsequent analyses. Differences in fold changes of ≥ 1.2 or ≤ 0.80 between patients with AD and controls were selected as significant miRNAs; we thus identified 29 upregulated and 31 downregulated miRNAs in the AD group (all *Q* < 0.05 with FDR correction, Fig. 2).

### Establishment of the predictive model

**Fig. 3:**
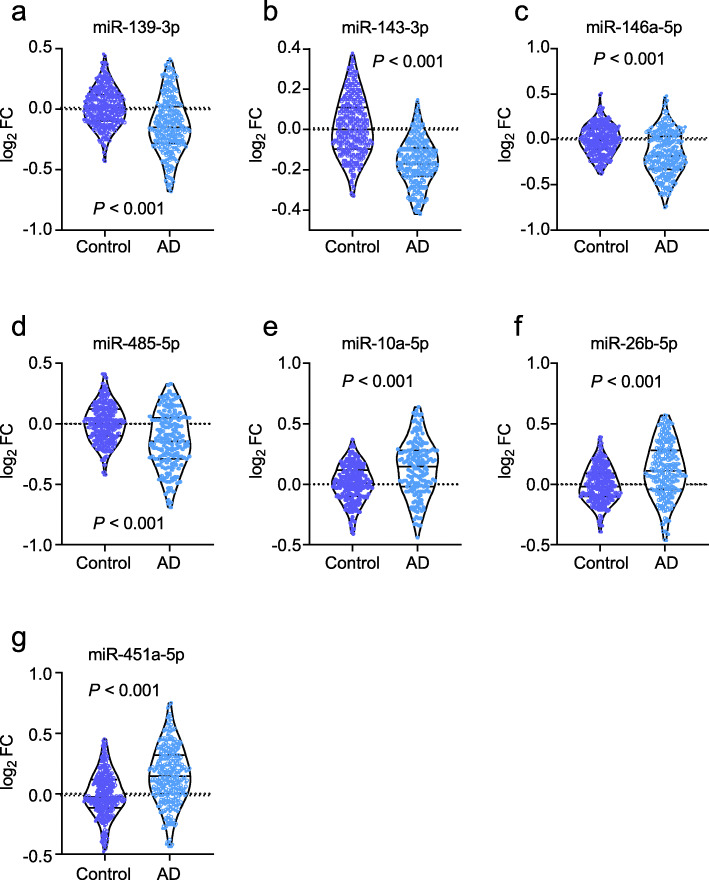
The measurements of miRNAs in Dataset 2. miR-139-3p (**a**), miR-143a-3p (**b**), miR-146a-5p (**c**), and miR-485-5p (**d**) were decreased in patients with Alzheimer’s disease (AD), and miR-10a-5p (**e**), miR-26b-5p (**f**), and miR-451a-5p (**g**) were increased in AD. Abbreviations: FC, fold change

**Fig. 4:**
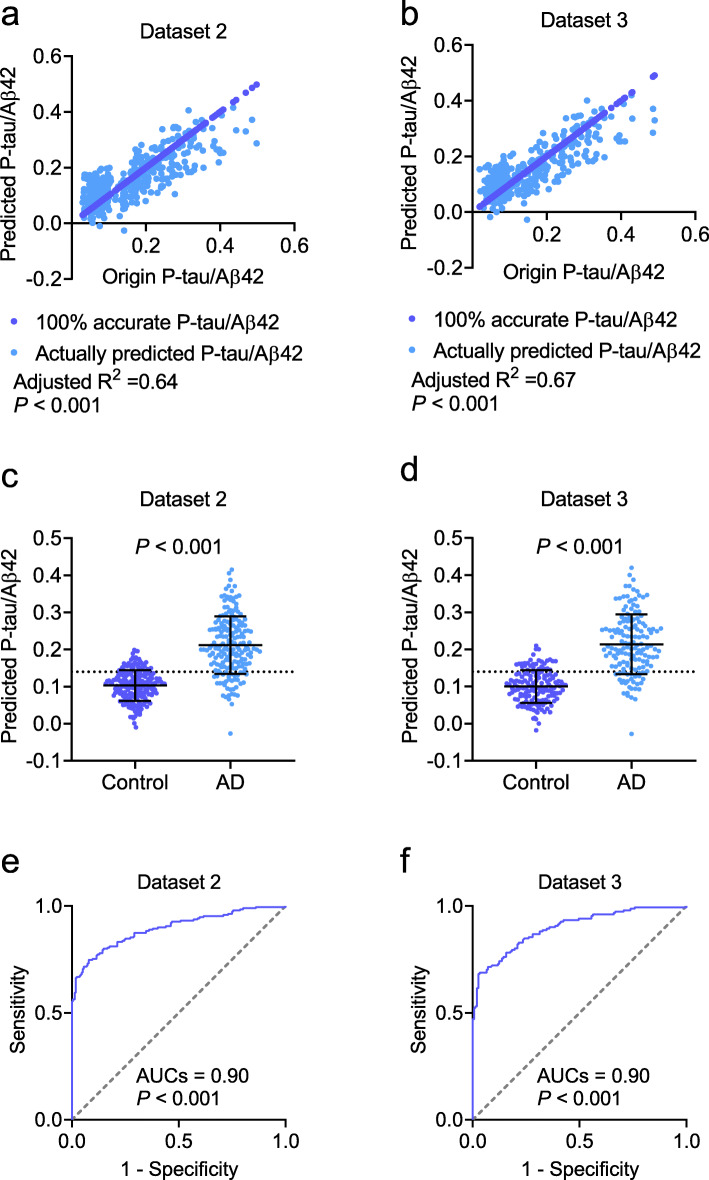
The establishment of the predictive model. The panel of the seven serum miRNAs was linearly correlated with the P-tau/Aβ42 ratio in CSF and predicted the P-tau/Aβ42 ratios in the patients with AD in Datasets 2 (**a**) and 3 (**b**). The predicted P-tau/Aβ42 was significantly increased in patients with AD in Datasets 2 (**c**) and 3 (**d**) and performed well in differentiating patients with AD from controls in Datasets 2 (**e**) and 3 (**f**). Abbreviations: AUC, area under the curve; AD, Alzheimer’s disease. The dotted line in c and d is cutoff value (0.14)

The extended samples (Dataset 2) were included to investigate the miRNAs that were differentially expressed between the patients with AD and controls. All 29 upregulated and 31 downregulated miRNAs in Dataset 1 were confirmed in Dataset 2, supporting the significance of the sequencing data obtained in the pilot study. We then analyzed the potential association between miRNA expression and the ratio of P-tau/Aβ42. The 29 upregulated and 31 downregulated miRNAs were included as independent variables in the linear regression model, and P-tau/Aβ42 as the dependent variable. After adjusting for age, sex, and ApoE ε4, the P-tau/Aβ42 ratio was found to be associated with a panel of seven miRNAs: miR-139-3p, miR-143-3p, miR-146a-5p, miR-485-5p, miR-10a-5p, miR-26b-5p, and miR-451a-5p (Fig. 3). Among these, miR-139-3p, miR-143-3p, miR-146a-5p, and miR-485-5p were decreased in patients with AD, while miR-10a-5p, miR-26b-5p, and miR-451a-5p were increased (*P* < 0.001; Fig. 3a–g). The linear regression analysis yielded *P* values of > 0.05 for the variables of age, sex, and ApoE ε4, indicating that the linear regression model was independent of these variables. Performing a linear regression analysis of only the panel of seven miRNAs, we found that the seven miRNAs were significantly associated with the values of the P-tau/Aβ42 ratio (adjusted *R*^2^ = 0.64, *P* < 0.001, Fig. 4a). The predictive equation of P-tau/Aβ42 was established and showed in Additional file 2: materials and methods. The equation was applied to Datasets 3 and 4 to predict P-tau/Aβ42 in the following analyses. We performed analyses to estimate the multicollinearity between the seven miRNAs in AD and controls. All tolerances were > 0.1, VIFs were < 10, eigenvalues were > 0, and condition index < 30, indicating that there is no significant multicollinearity between each miRNA. By applying the linear regression model, the predicted values of P-tau/Aβ42 ratio in patients with AD and the controls were calculated (Fig. 4c). Further ROC analyses showed that the P-tau/Aβ42 ratio predicted from the panel of seven miRNAs had a significantly high area under the curve (AUC; 0.90, *P* < 0.001; Fig. 4e) that far exceeded random chance (AUC of 50%).

### Confirmation of the prediction model

**Fig. 5:**
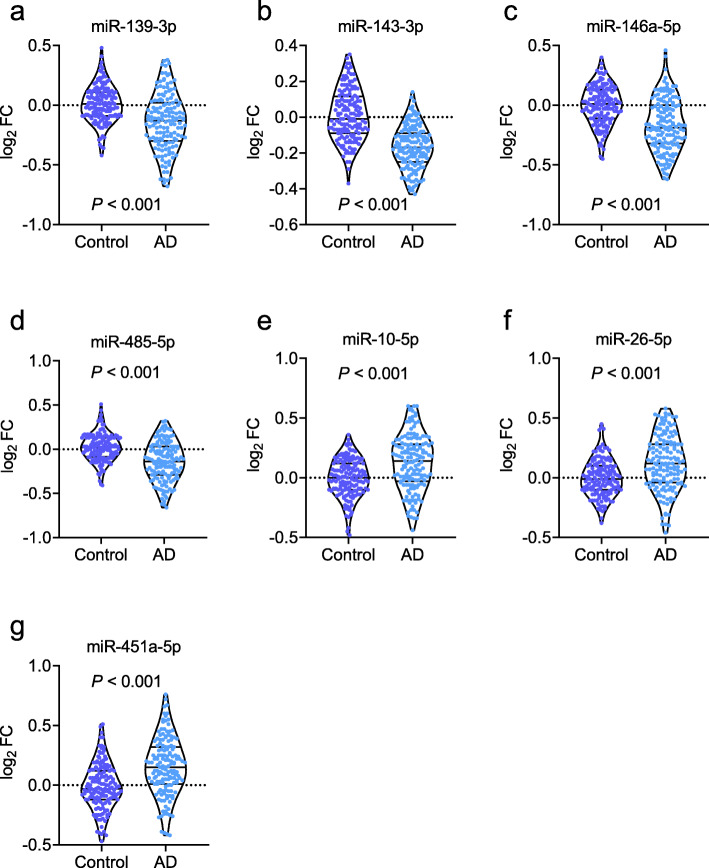
The measurements of miRNAs in Dataset 3. miR-139-3p (**a**), miR-143a-3p (**b**), miR-146a-5p (**c**), and miR-485-5p (**d**) were decreased in patients with Alzheimer’s disease (AD), and miR-10a-5P (**e**), miR-26b-5p (**f**), and miR-451a-5p (**g**) were increased. Abbreviations: FC, fold change

An independent dataset (Dataset 3) was used to confirm the above findings. We found that miR-139-3p, miR-143-3p, miR-146a-5p, and miR-485-5p were decreased in patients with AD relative to the controls, while miR-10a-5p, miR-26b-5p, and miR-451a-5p were increased in patients with AD (*P* < 0.001; Fig. 5a–g). Multicollinearity analyses showed that there is no significant multicollinearity between each miRNA in AD and controls (all tolerances > 0.1, VIFs < 10, eigenvalues > 0, and condition index < 30). The predictive equation generated from Dataset 2 was used to predict P-tau/Aβ42. The predicted P-tau/Aβ42 was highly associated with the actual P-tau/Aβ42 ratio in CSF (adjusted *R*^2^ = 0.67, *P* < 0.001, Fig. 4b). By using the predicted P-tau/Aβ42 ratio to distinguish the controls and AD (Fig. 4d), ROC analyses revealed a very high AUC (0.90, *P* < 0.001; Fig. 4f), which was the same as the AUC calculated from Dataset 2. Taken together, our model generated by the panel of 7 miRNAs in the blood may help to predict the P-tau/Aβ42 ratio in CSF, and diagnose of AD.

### Application of the prediction model

**Fig. 6:**
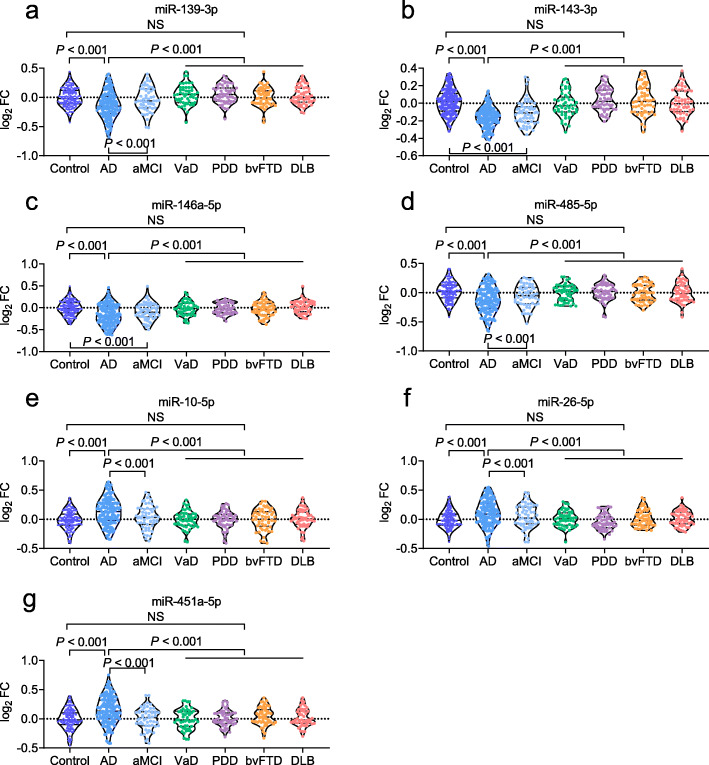
The measurements of miRNAs in AD, aMCI, VaD, PDD, bvFTD, and DLB. miR-139-3p (**a**), miR-143a-3p (**b**), miR-146a-5p (**c**), miR-485-5p (**d**), miR-10a-5P (**e**), miR-26b-5p (**f**), and miR-451a-5p (**g**) were measured. Abbreviations: AD, Alzheimer’s disease; aMCI, amnestic mild cognitive impairment; VaD, vascular dementia; PDD, Parkinson disease dementia; bvFTD, behavioral variant frontotemporal dementia; DLB, dementia with Lewy body; FC, fold change; NS, no significance

**Fig. 7:**
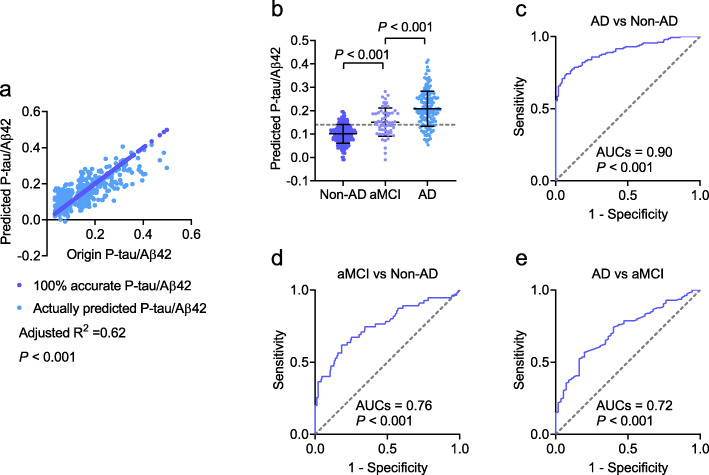
The application of the predictive model to patients with AD, aMCI, VaD, PDD, bvFTD, and DLB. The linear regression analyses were performed in patients with AD, aMCI, VaD, PDD, bvFTD, and DLB (**a**). By applying model, the predicted P-tau/Aβ42 ratio in CSF was compared between AD, aMCI, and non-AD subjects (combination of controls, VaD, PDD, bvFTD, and DLB) (**b**). The predicted P-tau/Aβ42 ratio successfully differentiated patients with AD from non-AD (**c**). The predicted P-tau/Aβ42 ratio was also compared between aMCI and Non-AD (**d**) and AD and aMCI (**e**). Abbreviations: AD, Alzheimer’s disease; aMCI, amnestic mild cognitive impairment; VaD, vascular dementia; PDD, Parkinson disease dementia; bvFTD, behavioral variant frontotemporal dementia; DLB, dementia with Lewy body; AUC, area under the curve

To assess the diagnostic capacity of the model when applying to subjects in clinical practice that may include controls, AD, and other neurodegenerative diseases, such as aMCI, VaD, PDD, bvFTD, and DLB, a fourth dataset was used. We obtained similar results to Datasets 1, 2 and 3; levels of miR-139-3p, miR-143-3p, miR-146a-5p, and miR-485-5p were decreased, while miR-10a-5p, miR-26b-5p, and miR-451a-5p were increased in patients with AD (*P* < 0.001; Fig. 6a–g). For aMCI, although all the seven miRNAs were altered, the alteration of miRNAs was slight in aMCI than in AD or in controls. This is reasonable, since aMCI is considered to be the early stage of AD. Thus, miRNAs may start changing at this stage, with changes in miRNAs in the AD stage becoming more significant as the disease progresses. All the seven miRNAs were not altered in patients diagnosed with VaD, PDD, bvFTD, and DLB (all *P* > 0.05), suggesting that these miRNAs are AD-specific. The predictive equation generated from Dataset 2 was used to predict P-tau/Aβ42. The predicted P-tau/Aβ42 was highly associated with the actual P-tau/Aβ42 ratio in CSF (adjusted *R*^2^= 0.62, *P* < 0.001, Fig. 7a). The predicted P-tau/Aβ42 ratio in AD patients was robustly increased compared to non-AD (combination of controls, VaD, PDD, bvFTD, and DLB) (*P* < 0.001, Fig. 7b). Further ROC analysis showed a very high AUC (0.90, *P* < 0.001, Fig. 7c), indicating that the panel of seven miRNAs is highly effective to identify AD from healthy controls and other neurodegenerative diseases. In addition, the predicted P-tau/Aβ42 ratio was also compared between aMCI and Non-AD (0.76, *P* < 0.001, Fig. 7d), and AD and aMCI (0.72, *P* < 0.001, Fig. 7e).

## Discussion

The present study identified an association between a panel of blood miRNAs and the ratio of P-tau/Aβ42 in the CSF of patients with AD, suggesting miRNAs as a promising tool for predicting the Aβ42 and P-tau levels in patients with AD.

Biomarkers have played an important role in the diagnosis [1] and research [17] of AD. Because of its minimal invasiveness and relatively low cost, the use of peripheral blood to diagnose AD has garnered increasing attention. The attendant surge in research has revealed a series of promising markers in the blood, including Aβ42 [34], the neurofilament light protein (NFL) [35], P-tau181 and 217 [36], exosomal Aβ42, T-tau, P-tau, synaptic proteins, and inflammatory factors [10, 11, 37]. Despite their high diagnostic efficacy, this method is subject to limitations. Requiring advanced skill and specialized equipment, the collection and measurement of Aβ42 from the blood by immunoprecipitation coupled with mass spectrometry is cost-prohibitive. Moreover, NFL is not a specific biomarker for AD; aberrant NFL concentrations may indicate other diseases causing axonal damage, such as multiple sclerosis (MS) [38], frontotemporal dementia (FTD) [39], and amyotrophic lateral sclerosis (ALS) [40]. While blood P-tau can be easily measured, it requires a specialized testing system that may require further development before its cost can allow for extensive, wide-spread use [41]. The screening of biomarkers from exosomes in the blood may be excessively expensive, as it requires the collection and enrichment of neuron-derived exosomes through a series of experiments, including immunoprecipitation and ELISA.

By contrast, the analysis of miRNAs in blood is an antibody-independent and easily implemented method for differentiating patients with AD from their cognitively normal counterparts, as well as patients with other forms of dementia. By only requiring the widely used technique of qPCR to quantify a panel of serum miRNAs, our technique can predict the P-tau/Aβ42 ratio—a well-known AD biomarker—in the CSF. To the best of our knowledge, this study is the first to provide support for an association between miRNAs in the blood with P-tau/Aβ42 in CSF and is a promising application to screen for AD in older populations at relatively little cost and with minimal invasiveness.

Recent studies have increasingly implicated miRNAs in AD pathology; miRNAs regulate the expression of APP [42–45] and proteins involved in APP metabolism, such as α-secretase, ADAM10 [46, 47], β-secretase, and BACE1 [48, 49]. miRNAs also play an important role in Aβ clearance, e.g., miRNAs can downregulate ApoE lipidation [50] and TREM2 levels [51] and impair Aβ metabolism in the brain. Moreover, miRNA levels are related to the expression and hyperphosphorylation of tau in the brain [52–54] and are involved in other AD-associated mechanisms, such as aberrant mitochondrial function [55–57], autophagy [58, 59], mitophagy [60, 61], neurotransmitter release and clearance [62, 63], and synaptic plasticity [64].

Due to their important roles in the pathology of AD, miRNAs can act as biomarkers of the disease [65]. miRNAs have been used as biomarkers for a range of diseases, such as cancer [66, 67], cardiovascular disease [68, 69], and diabetes [70, 71]. In agreement with the observations of dysregulation of miRNAs in the CSF of patients with AD [72], alterations of miRNAs in the peripheral blood have shown potential as promising candidate biomarkers of AD. The combination of several miRNAs was able to discriminate the CSF of patients with AD from that of controls with sufficient accuracy [73]. A recent literature review showed that, among 137 miRNAs found to be abnormally expressed in the blood of patients with AD, 36 had been replicated independently in more than one study [74]. This finding provides evidence in support of the use of miRNAs as diagnostic biomarkers. Moreover, a signature of 12 miRNAs in the blood could not only inform the discrimination between AD patients and controls but also between patients with AD and those with other neurological disorders, such as Parkinson’s disease and schizophrenia [14]. While our findings confirm the utility of miRNAs as biomarkers for AD, our study further suggests that miRNAs could reflect the P-tau/Aβ42 ratio in the CSF, an established AD biomarker. We attribute this association to the important roles of miRNAs in the regulation of AD pathways in the brain. We further compared miRNA levels between AD and VaD, PDD, bvFTD, and DLB. Although all these degenerative diseases have some similar clinical manifestations such as cognitive impairment, AD has its unique pathological process, which may be the reason why the changes of these seven miRNAs are AD-specific and differentiates AD from other neurodegenerative diseases. However, our results concerning the upregulation or downregulation of a single miRNA were inconsistent with the observations of other studies: while miR-17 was reported to be significantly altered in the blood of AD patients [75], our own study could not confirm this. This discrepancy might suggest that miRNA expression may vary according to ethnicities. Further multi-center studies are needed to evaluate genetic differences in miRNA expression between different ethnic populations.

This study is limited by its cross-sectional design. Although we confirmed that a panel of seven miRNAs could be applied as diagnostic biomarkers of AD, longitudinal designs would be better suited for the evaluation of the performance of these biomarkers. Hence, longitudinal studies investigating the relationship between the levels of biomarkers and the decline in cognitive functions of patients are warranted. This study was further limited by its not having considered patients with mild cognitive impairment that progressed to either AD or stable amnestic mild cognitive impairment. The application of our method to the prediction of the progression from prodromal to probable AD is thus diminished. Finally, measuring miRNAs with qPCR is a relative quantification approach that cannot indicate absolute levels of miRNAs in the blood, limiting the comparisons of the absolute levels of miRNA between our study and others.

## Conclusions

In summary, the results of the present study indicate that a panel of seven miRNAs are potential blood biomarkers for AD. Specifically, the association between the levels of seven miRNAs and the P-tau/Aβ42 ratio in the CSF of AD patients confirms that miRNA biomarkers may reflect pathological changes in the brain and, therefore, can inform the identification of patients with AD. However, our findings require further validation in longitudinal studies.

## Supplementary Information

**Additional file 1: Figure S1.** The flowcharts of the screening for subjects in Dataset 1

**Additional file 2: Table S1.** List of the primers for real-time quantitative PCR. **Table S2.** Coefficient of variation of qPCR. **Table S3.** ELISA kits information. **Materials and methods**

## Data Availability

The datasets used and/or analyzed during the current study are available from the corresponding author on reasonable request.

## Abbreviations

AD: Alzheimer’s disease
Aβ: Amyloid β
ApoE: Apolipoprotein E
APP: Amyloid precursor protein
AUC: Area under the curve
BACE1: Beta-site APP cleaving enzyme 1
bvFTD: Behavioral variant frontotemporal dementia
CSF: Cerebrospinal fluid
DLB: Dementia with Lewy body
ELISA: Enzyme-linked immunosorbent assay
miRNA: MicroRNAs
MMSE: Mini-Mental State Examination
NIA-AA: National Institute on Aging and Alzheimer’s Association
PDD: Parkinson disease dementia
PET: Positron emission tomography
P-tau: Phosphorylated-tau
qPCR: Quantitative real-time PCR
ROC: Receiver operating characteristic
T-tau: Total-tau
UTR: Untranslated region
VaD: Vascular dementia
