# Supplementary figures and images for: Prediction of P-tau/Aβ42 in the cerebrospinal fluid with blood microRNAs in Alzheimer’s disease
Source: BMC Med. 2021 Nov 15;19:264. doi: 10.1186/s12916-021-02142-x (PMC8591889; doi:10.1186/s12916-021-02142-x)

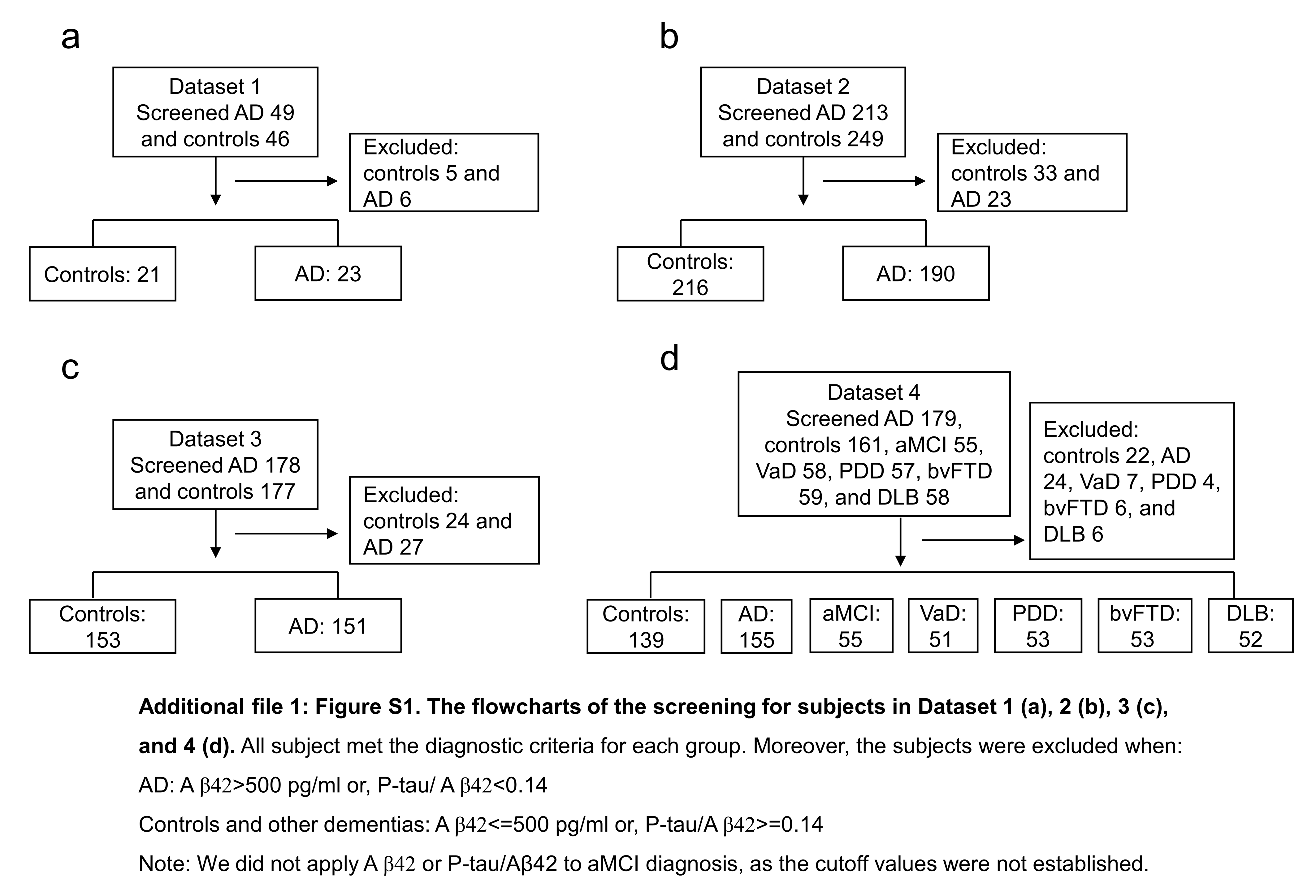

Supplement: Supplementary file 1 — Additional file 1: Figure S1. The flowcharts of the screening for subjects in Dataset 1 [file 12916_2021_2142_MOESM1_ESM.tif]
