# Supplementary material for: Prediction of P-tau/Aβ42 in the cerebrospinal fluid with blood microRNAs in Alzheimer’s disease
Source: BMC Med. 2021 Nov 15;19:264. doi: 10.1186/s12916-021-02142-x (PMC8591889; doi:10.1186/s12916-021-02142-x)
Supplement: Supplementary file 2 — Additional file 2: Table S1. List of the primers for real-time quantitative PCR. Table S2. Coefficient of variation of qPCR. Table S3. ELISA kits information. Materials and methods [file 12916_2021_2142_MOESM2_ESM.docx]

**Supplementary Material**

**Title:**

**Prediction of P-tau/Aβ42 in the cerebrospinal fluid with blood microRNAs in Alzheimer’s disease**

Longfei Jia, et al.

The Supplementary materials include the following information:

Supplementary Table 1. List of the primers for real-time quantitative PCR

Supplementary Table 2. Coefficient of variation of qPCR

Supplementary Table 3. ELISA kits information

Supplementary materials and methods

**Supplementary Table 1. List of the primers for real-time quantitative PCR**

| **microRNA/reference** | **Sequence** |
| --- | --- |
| hsa-miR-10a-5p | CTGTAGATCCGAATTTGTGA |
| hsa-miR-26b-5p | TTCAAGTAATTCAGGATAGGTAAAA |
| hsa-miR-139-3p | GCGGCCCTGTTGGAGTAAA |
| has-miR-143-3p | TGAGATGAAGCACTGTAGCTCAAA |
| hsa-miR-146a-5p | AGAACTGAATTCCATGGGTTAA |
| hsa-miR-451a-5p | AAACCGTTACCATTACTGAGTTAA |
| hsa-miR-485-5p | CTGGCCGTGATGAATTCAAAA |
| cel-miR-39-3p | UCACCGGGUGUAAAUCAGCUUG |

**Supplementary Table 2. Coefficient of variation of qPCR**

| **microRNA** | **CV% of Dataset 2** | **CV% of Dataset 3** | **CV% of Dataset 4** |
| --- | --- | --- | --- |
| hsa-miR-10a-5p | 0.69-3.72 | 0.71-3.73 | 0.86-2.97 |
| hsa-miR-26b-5p | 0.83-3.65 | 0.72-3.42 | 0.58-2.92 |
| hsa-miR-139-3p | 0.83-3.77 | 0.76-3.86 | 0.88-3.49 |
| has-miR-143-3p | 0.88-3.68 | 0.87-3.62 | 0.98-3.59 |
| hsa-miR-146a-5p | 0.62-3.74 | 0.84-3.67 | 0.83-3.32 |
| hsa-miR-451a-5p | 0.83-3.39 | 0.75-3.78 | 0.68-3.70 |
| hsa-miR-485-5p | 0.82-3.89 | 0.78-3.77 | 0.86-3.25 |

To improve experimental precision, triplicates for qPCR were performed. Coefficient of variation (CV) was calculated using standard deviation divided by mean value of a group of replicates. All CV% in the study were lower than 5%, indicating that the data were of high quality.

**Supplementary Table 3. ELISA kits information**

| **ELISA kits** | **Catalog numbers** | **Assay ranges (pg/ml)** | **Current data ranges (pg/ml)** | |
| --- | --- | --- | --- | --- |
| **Aβ42** |  |  | |  |
| INNOTEST (Japan） | 81576 | 62.5-4000 | | 182-1052 |
| **T-tau** |  |  | |  |
| INNOTEST (Japan） | 81572 | 34-2500 | | 51-1079 |
| **P-T181-tau** |  |  | |  |
| INNOTEST (Japan) | 81581 | 15.6-1000 | | 22-139 |

**Supplementary materials and methods**

**Diagnostic criteria**

**AD diagnosis [1]:**

Inclusion criteria:

- - 1. The cognitive impairment involves a minimum of two of domains (memory functions，executive function，visuospatial abilities， language function and behavior), which interferes with the ability to function at work or in activities of daily living.
    2. Insidious onset (symptoms exhibit a gradual onset over months to years, not sudden over hours or days)
    3. Meeting the cutoff values of MMSE, CDR, P-tau/ Aβ42, and MRI (shown in table 1 below).

Exclusion criteria:

- - 1. Delirium or psychiatric disorders.
    2. Substantial concomitant cerebrovascular disease, or the presence of multiple or extensive infarcts or severe white matter hyperintensity burden.
    3. Other types of dementia (dementia with Lewy bodies, behavioral variant frontotemporal dementia, semantic variant primary progressive aphasia or non-fluent/agrammatic variant primary progressive aphasia)
    4. Other neurological diseases, or a non-neurological medical comorbidity or use of medications that could have a substantial effect on cognition.

**aMCI diagnosis [2]:**

- - 1. Memory complaint, preferably corroborated by an informant.
    2. Memory impairment relative to age-matched and education-matched healthy individuals.
    3. Typical general cognitive function.
    4. Largely intact activities of daily living.
    5. Not clinically demented.
    6. Meeting the cutoff values of MMSE, CDR, MRI.

We did not apply the P-tau/Aβ42 to aMCI diagnosis, as the cutoff values were not established.

**Normal controls:**

- - 1. Have normal cognitive assessment.
    2. Have normal MRI, P-tau/Ab42 and T-tau/Aβ42.
    3. Did not have tumors, small vascular disease, white matter lesions, or other neurological diseases.

|  | MMSE | CDR | P-tau/Aβ42 | MRI |
| --- | --- | --- | --- | --- |
| normal | ≥28 | 0 | ≤0.14 | normal |
| aMCI | <24 and ≤27 | 0 or 0.5 | - | MTA: 0-1. Excluding: tumour, small vascular disease, white matter lesions. |
| AD | ≤24 | ≥1 | >0.14 | MTA: 2-3. Excluding: tumor, small vascular disease, white matter lesions. |

Table 1. The cutoff values of MMSE, CDR, P-tau/ Aβ42, and MRI used to diagnose AD and aMCI.

**VaD diagnosis [3]:**

Meet all of the following:

(1) Dementia

- - 1. Impairments of two or more cognitive domains.
    2. Exclusion of other diseases such as consciousness, delirium, psychosis, severe aphasia

(2) Cerebrovascular disease (CVD)

- - 1. Presence of focal signs on neurologic examination
    2. Evidence of CVD by brain CT or MRI imaging

(3) A relationship between the above two disorders (meet at least one of the following)

- - 1. Onset of dementia within three months following a recognized stroke;
    2. Abrupt deterioration in cognitive functions; or fluctuating, stepwise progression of cognitive deficits.

**PDD diagnosis [4]:**

- - 1. Core Features:

1. Diagnosis of Parkinson’s disease.

2. A dementia syndrome develops (more than one cognitive domain impaired; daily life abilities impaired). The decline develops after Parkinson's symptom from premorbid level

- - 1. Must meet all core features.
    2. No factors suggesting other diagnoses.

**bvFTD diagnosis [5]:**

1. At list three of the following behavioural/cognitive symptoms:
   - 1. Progressive deterioration of behaviour and/or cognition
     2. Early behavioural disinhibition
     3. Early apathy or inertia
     4. Early loss of sympathy or empathy
     5. Early perseverative, stereotyped or compulsive/ritualistic behaviour
     6. Hyperorality and dietary changes
     7. Neuropsychological profile: executive/generation deficits with relative sparing of memory and visuospatial functions

(2) All of the following symptoms:

- - 1. Meets criteria in (1)
    2. Exhibits significant functional decline
    3. Imaging results: Frontal and/or anterior temporal atrophy on MRI or CT; Frontal and/or anterior temporal hypoperfusion or hypometabolism on PET

**DLB diagnosis [6]:**

At list two of the following behavioural/cognitive symptoms:

- - 1. Fluctuating cognition with pronounced variations in attention and alertness
    2. Recurrent visual hallucinations that are typically well formed and detailed
    3. REM sleep behavior disorder, which may precede cognitive decline
    4. One or more spontaneous cardinal features of parkinsonism

**Methods**

The measurements of 7-micrRNA panel in Dataset 2 were used to establish the predictive model of P-tau/Aβ42. The equation was P-tau/Aβ42 =0.106 - 0.068 × miR-146a-5p - 0.093 × miR-139-3p - 0.093 × miR-485-5p + 0.089 × miR-26-5p + 0.079 × miR-451a-5p + 0.099 × miR-10-5p - 0.203 × miR-143-3p. This equation was applied to Dataset 3 and 4.

1. Jack CR, Jr., Bennett DA, Blennow K, Carrillo MC, Dunn B, Haeberlein SB, Holtzman DM, Jagust W, Jessen F, Karlawish J *et al*: **NIA-AA Research Framework: Toward a biological definition of Alzheimer's disease**. *Alzheimers Dement* 2018, **14**(4):535-562.

2. Gauthier S, Reisberg B, Zaudig M, Petersen RC, Ritchie K, Broich K, Belleville S, Brodaty H, Bennett D, Chertkow H *et al*: **Mild cognitive impairment**. *Lancet* 2006, **367**(9518):1262-1270.

3. Roman GC, Tatemichi TK, Erkinjuntti T, Cummings JL, Masdeu JC, Garcia JH, Amaducci L, Orgogozo JM, Brun A, Hofman A *et al*: **Vascular dementia: diagnostic criteria for research studies. Report of the NINDS-AIREN International Workshop**. *Neurology* 1993, **43**(2):250-260.

4. Goetz CG, Emre M, Dubois B: **Parkinson's disease dementia: definitions, guidelines, and research perspectives in diagnosis**. *Ann Neurol* 2008, **64 Suppl 2**:S81-92.

5. Rascovsky K, Hodges JR, Knopman D, Mendez MF, Kramer JH, Neuhaus J, van Swieten JC, Seelaar H, Dopper EG, Onyike CU *et al*: **Sensitivity of revised diagnostic criteria for the behavioural variant of frontotemporal dementia**. *Brain* 2011, **134**(Pt 9):2456-2477.

6. McKeith IG, Boeve BF, Dickson DW, Halliday G, Taylor JP, Weintraub D, Aarsland D, Galvin J, Attems J, Ballard CG *et al*: **Diagnosis and management of dementia with Lewy bodies: Fourth consensus report of the DLB Consortium**. *Neurology* 2017, **89**(1):88-100.
